# Effects of Vitamin D Deficiency on the Function of the Cardiac Autonomic Nervous System in Rats

**DOI:** 10.1155/2022/4366948

**Published:** 2022-03-23

**Authors:** Xuemei Luo, Jie Xiong, Hong Cai, Runmei Zou, Fang Li, Yuwen Wang, Cheng Wang

**Affiliations:** Department of Pediatric Cardiovasology, Children's Medical Center, The Second Xiangya Hospital, Central South University, Changsha, Hunan, China

## Abstract

**Background:**

Previous studies have shown that autonomic nervous system (ANS) dysfunction was closely related to vitamin D (VD) deficiency, but the mechanism remained unclear. The purpose of this study was to evaluate the mechanism of VD^def^ on the function of cardiac ANS in rats.

**Methods:**

After 10 weeks of VD deficiency feeding, we successfully established a VD-deficient rat model. The body weight of rats was recorded, and the levels of calcium (Ca), phosphorus (P), creatinine (CRE), triglyceride (TG), hemoglobin (HG), and 25(OH)VD3 in serum were detected by corresponding kits. Short-time frequency domain analysis was used to evaluate the heart rate variability (HRV) of all rats. The expression of tyrosine hydroxylase (TH) in the atria and ventricle were detected by IHC. ELISA was used to determine the levels of acetyl choline (Ach) and nitric oxide (NO). HPLC was used for the detection of norepinephrine (NE). The expressions of KIR3.1, HERG, KVLQT1, and Mink were detected by qRT-PCR and western blot.

**Results:**

After 10 weeks of VD deficiency feeding, serum 25(OH)VD3 levels were markedly reduced in the VD^def^ group, and sera Ca and P, as well as body weight, were notably decreased in the VD^def^ group. In resting and motion states, VD deficiency resulted in a decline in HF levels and a mark increase in VLF and LF/HF levels. VD deficiency caused a reduction in the release of the local cardiac neurotransmitters TH and Ach. NE and NO levels were also remarkably depressed in the VD^def^ group. In addition, VD deficiency resulted in severely impaired expression of potassium channel proteins.

**Conclusion:**

VD deficiency leads to cardiac ANS dysfunction. The imbalance in heart rate variability, impaired release and secretion of neurotransmitters and local plasma hormones in the heart, and downregulation of potassium channel protein expression caused by VD deficiency may be closely related to this dysfunction.

## 1. Introduction

The autonomic nervous system (ANS), also known as the vegetative nervous system (VNS), is an integral part of the peripheral efferent nervous system that regulates the activity of visceral and vascular smooth muscle, cardiac muscle, and glands [1]. It mainly comprised two portions, the sympathetic nervous system (SNS) and the parasympathetic nervous system (PNS), and has extensive innervation to almost all organ systems in the human body [2–4]. The cardiac is an organ receiving dual innervation from the SNS and PNS branches of the ANS [5]. Cardiac ANS control is a dynamic process in both health and disease [6]. The close relationship between the cardiac and ANS was previously demonstrated in the 1970s, not only regulating cardiac rate and hemodynamic changes but also participating in the development of arrhythmia [7]. Previous studies have shown that the main cause of the dysfunction of ANS is the imbalance between the SNS and the PNS. ANS dysfunction will bring patients' physical discomfort, psychological discomfort, mental discomfort, and other negative effects [8]. Therefore, maintaining the balance of ANS is a major challenge facing current research.

VitaminD (VD) deficiency and insufficiency have become a worldwide health concern and a major burden on global public health care [9]. It afflicts hundreds of millions of children and adults due to its dramatically increasing prevalence [10]. VD deficiency directly or indirectly affects a variety of acute and chronic diseases such as autoimmune diseases, cardiovascular diseases, and neurological disorders [11, 12]. Currently, a large number of studies have shown an important association between VD deficiency and ANS activity. For example, Chaudhari et al. found that patients with postural standing tachycardia syndrome had lower levels of ossified triol (1,25(OH)D_3_) than normal subjects and that appropriate supplementation improved patient symptoms, revealing that VD inhibits autonomic nervous system dysfunction in the body [13]. A clinical study reported that cardiac autonomic function was impaired in people with VD deficiency despite the absence of overt cardiac involvement and symptoms [14]. It has been proposed that VD deficiency may be a trigger for cardiac autonomic dysfunction in children and adolescents with vasovagal syncope [15]. These studies suggest that VD plays a crucial role in the maintenance of cardiac autonomic homeostasis.

Although we know that there is an important relationship between VD deficiency and ANS, the exact mechanism between the two is not clear. Therefore, this study established a rat model of VD deficiency to explore the possibility that VD deficiency may affect the function of cardiac ANS in rats by altering neurotransmitters and ion channel proteins. This will provide some basis for the experimental study of VD deficiency and cardiac ANS function and its further clinical application.

## 2. Materials and Methods

### 2.1. Establishment of the VD^def^ Rat Model

Thirty 9 to 10-week-old male SD rats weighing 275-300 g were used in the laboratory, which were provided by Hunan SJA Laboratory Animal Co., Ltd. Thirty rats were randomly divided into the VD^def^ (VD deficient) and control groups of 15 rats each. VD^def^ rats were fed a VD-deficient chow for 10 weeks, and control rats were fed an adequately formulated chow for the same period. After 10 weeks of feeding, heart rate variability was assessed at rest and motion status. Rats were then killed and cardiac effluent; left/right atria and ventricles were collected from the rats for subsequent experiments. The animal use protocol listed below has been reviewed and approved by the Animal EthicsThe Second Xiangya Hospital, Central South University (approval number: 2021112).The treatment of animals during the experiment conforms to the standards of "Guiding Opinions on Being kind to Experimental Animals" issued by the Ministry of Science and Technology in 2006.

### 2.2. Detection of Physiological and Biochemical Indexes in Rats

We used electronic balance (YB2000N, ZMSC, China) to weigh the weight of rats in each group. During the feeding period, the rats were weighed once a week and the data were recorded. For biochemical determination, blood was collected during euthanasia. Serum was obtained by centrifugation at 3500 rpm for 10 minutes. Next, we performed Ca assay kit (C004-2-1, Nanjing Jiancheng Institute of Biological Engineering, Nanjing, China), inorganic phosphorus (P) assay kit (C006-1-1, Nanjing Jiancheng Institute of Biological Engineering, Nanjing, China), creatinine (CRE) assay kit (C011-2-1, Nanjing Jiancheng Institute of Biological Engineering, Nanjing, China), triglyceride (TG) assay kit (A110-1-1, Nanjing Jiancheng Institute of Biological Engineering, Nanjing, China), hemoglobin (HG) assay solution instructions (C021-1-1. Nanjing Jiancheng Institute of Biological Engineering, Nanjing, China), and 25(OH)VD_3_ assay kit (SEKM-0140, Solarbio) to determine the concentrations of Ca, P, CRE, TG, HG, and 25(OH)VD3.

### 2.3. HRV Assessment

Since the results of HRV will be affected by many factors, we unified the parameters in the detection [16, 17]. We used short-term frequency domain analysis to analyze the heart rate variability HRV of VD^def^ rats and vitamin sufficient rats in resting state and exercise stimulation state [14]. The length of the analyzed recording was 30 min. ECG acquisitions were performed on all rats at week 11 of the experiment. The acquisition rate was 400 Hz. The fixed time step was 0.2 s. The scan speed was 50 ms/div. Sensitivity was 1 mV. All subjects recorded 24-hour ambulatory ECG with cardioscan Holter ECG system and three-channel analog recorder. The dynamic ECG analysis system was used to analyze the ECG data and calculate the HRV values automatically. HRV frequency domain indexes include low-frequency power (LF, 0.04-0.15 Hz), high-frequency power (HF, 0.15-0.4 Hz), and very-low-frequency power (VLF, 0.003-0.04 Hz).

### 2.4. Immunohistochemistry (IHC)

The myocardial tissue of rats was taken to detect the tyrosine hydroxylase (TH). The fixed tissue sections of the slide were placed at room temperature according to the previously studied IHC method [18]. After baking the slices at 60°C for 12 h, the slices were placed in xylene for 20 min, 3 times. Then, they were treated with 100%, 95%, 85%, and 75% ethanol in turn for 5 min at each stage. The slices were soaked in distilled water for 5 min until dewaxing to water. Next, we heated and repaired the antigen. We dipped the slices in 0.01 M citrate buffer (pH 6.0) and turned off the power after boiling in the microwave. We removed them from the microwave after cooling for 23 min until cooled to room temperature. After cooling, we washed them with 0.01 M PBS (pH 7.2-7.6) for 3 min × 3 times and added 1% periodic acid and inactivated endogenous enzymes at room temperature for 10 minutes. We washed them with PBS for 3 min × 3 times. Then, we incubated the first antibody. We dropped appropriately diluted primary antibody TH (25859-1-AP, 1: 100, PTG) at 4°C overnight. We washed them with PBS for 5 min × 3 times. The secondary antibody was incubated by adding 50-100 *μ*L anti-rabbit-IgG antibody-HRP polymer and incubated at 37°C for 30 min, then washed with PBS for 5 min × 3 times. Finally, we used DAB to color them. We dropped the prepared color development agent DAB working solution of 50-100 *μ*L, incubated for 1-5 min at room temperature, controlled the reaction time under the mirror, and washed with distilled water. Hematoxylin was redyed for 5-10 min, washed with distilled water and then returned to blue with PBS. We dehydrated the slices with all levels of alcohol (60-100%) for 5 min per level. We removed the slices and placed it in xylene for 10 min, twice. Then, we sealed them with neutral gum and observed them under a microscope.

### 2.5. Enzyme Linked Immunosorbent Assay (ELISA)

20 mg of the heart tissue of rats was taken from each group of experimental samples. Then blood contamination was washed with PBS. We cut the heart tissue into small pieces, put it into a tissue grinder (homogenizer), and then add 200 *μ*L 1 × PBS was mixed to make it homogenate. Then, the tissue homogenate was centrifuged in a bench top high-speed freezing centrifuge (#H1650R, xylxjlab, China) for 5 minutes to remove the supernatant. The centrifuge was set at 2-8°C, 5000 rpm. We took the appropriate amount of supernatant for experiment immediately or separated and stored the supernatant at -20°C or -80°C. In order to avoid repeated freezing and thawing, the thawed samples should be tested after centrifugation again. Then, we detected the concentration of Ach and nitric oxide (NO) in a rat heart tissue according to the methods in the instructions of Ach kit (A105-1, Nanjing Jiancheng Bioengineering Institute, China) and NO kit (A013-2-1, Nanjing Jiancheng Bioengineering Institute, China). The quantification of Ach and NO was performed by a multifunctional enzyme marker (MB-530, HEAES, China).

### 2.6. High-Performance Liquid Chromatography (HPLC)

We used HPLC to determine the concentration of NE in sympathetic neurons [19]. We mixed 200 mg plasma, 800 *μ*L lysate buffer, 10% HCl, and 10 mM BPDS. Then, the homogenate was centrifuged at 14,000 rpm and at 4°C for 5 min. Next, the supernatant was recycled and injected into the chromatographic system. The mobile phase consisted of acetonitrile (1%) and 20 mM potassium dihydrogen phosphate buffer (pH 7.0) (99%) at a flow rate of 1 mL/min. NE was detected at 210 nm using a 2489 UV/Vis detector (Waters, MA).

### 2.7. Quantitative Real-Time PCR (qRT-PCR)

Total RNA was extracted from the left and right atria and left and right ventricle cells of rats using Trizol. The expressions of Kir3.1, HERG, KVLQT1, and Mink in cells were detected by fluorescence quantification. 5 *μ*L RNA was taken from each group and mixed with 6∗ loading buffer at a ratio of 1 : 5. Electrophoresis was performed under 140 V constant pressure. The results of electrophoresis were observed under a gel imaging system. The cDNA was reverse transcribed using tissue total mRNA as a template. The SYBR method was used to conduct RT-qPCR experiment. The sequence of the target gene was searched on NCBI, and primers were designed using Primer5 software. The primers were synthesized by Shanghai Sangon Biotech Co., Ltd. GAPDH was used as the internal reference to detect the expression level. The primer sequences are shown in Table 1.

### 2.8. Western Blot (WB)

In order to detect the expression of Kir3.1, HERG, KVLQT1, and Mink, proteins were extracted from the atrium and ventricle of rats according to the RIPA cleavage buffer instructions. Protein quantification was performed on the samples of each group according to the BCA protein assay kit. Loading buffer of SDS-PAGE was mixed, and the mixture was heated in boiling water bath at 100°C for 5 minutes. The protein was adsorbed on the PVDF membrane by gel electrophoresis and sealed with 5% skimmed milk solution at room temperature for 2 h. KVLQT1 (AB84819,1: 1000, Abcam), HERG (AB196301, 1: 1500, Abcam), Mink (13137-1-AP,1: 500, Proteintech), and GAPDH (60008-1-Ig, 1: 5000, Proteintech) were incubated at room temperature for 90 min. At room temperature, HRP goat anti-mouse IgG (SA00001-1, 1: 5000, Proteintech) was washed three times with TBST and incubated with HRP Goat Anti-Mouse IgG (SA00001-2, 1: 6000, Proteintech). ECL color exposure was analyzed using the Odyssey Infrared Imaging System (Li-Cor Biosciences, Lincoln, NE, USA) to detect protein bands. GAPDH was used as an internal reference to detect the expression level.

### 2.9. Statistical Analysis

Statistical analysis was performed using GraphPad 8.0 software, and three independent experimental data were expressed as the mean ± standard deviation (SD). Two-way ANOVA was used to compare the changes in rat body weight at different time points. Student's *t*-test was used to compare the bodyweight of rats between different groups at the same time point. The statistical analysis of other indexes between the control group and the VD^def^ group was performed using Student's *t*-test. *P* < 0.05 was considered statistically significant.

## 3. Results

### 3.1. The Changes of Physiological Indexes after the Establishment of VD^def^ Rat Model

**Figure 1:**
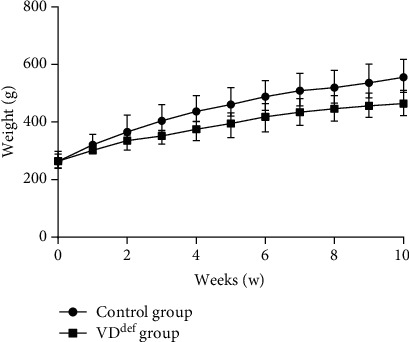
Effects of VD^def^ on body weight in rats. The red triangle represents the weight of the control group, and the blue triangle represents the weight of VD^def^ group. ^∗^*P* < 0.05 vs. the control group.

Over a period of 10 weeks of vitamin D deficiency feeding, the levels of 25(OH)VD3 in the serum of the VD deficiency group rats were significantly reduced. Serum levels of Ca and P were also markedly decreased in the VD deficiency group, whereas creatinine, triglyceride, and hemoglobin showed no statistically considerable differences in the rats of different treatments (Table 2). Through recording the body weight of the rats, we noticed that there was no apparent difference in the body weight of the rats in the two groups (Figure 1).

### 3.2. Effects of VD Deficiency on HRV in Rats

**Figure 2:**
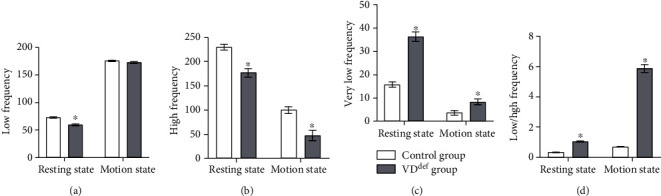
Effects of VD^def^ on HRV in rats. LF (a), HF (b), VLF (c), and LF/HF (d) were analyzed in resting state and motor stimulation state. ^∗^*P* < 0.05 vs. the control group. LF: low frequency; HF: high frequency; VLF: very low frequency.

We examined the effects of VD deficiency on heart rate variability (HRV) in rats at rest and in motion state, respectively. As shown in Figure 2(a), the LF of the VD-deficient group was significantly lower than that of the control group in the resting state, while there was no significant difference between the two groups in the motion state. In both resting state and motion state, HF was lower in the VD-deficient group than in the control group (Figure 2(b)). In contrast, the levels of VLF and LF/HF were obviously higher in the VD-deficient group than in the control group in both states (Figures 2(c) and 2(d)).

### 3.3. VD Deficiency Changes the Expression of Neurotransmitter in the Autonomic Nervous System of Rats

**Figure 3:**
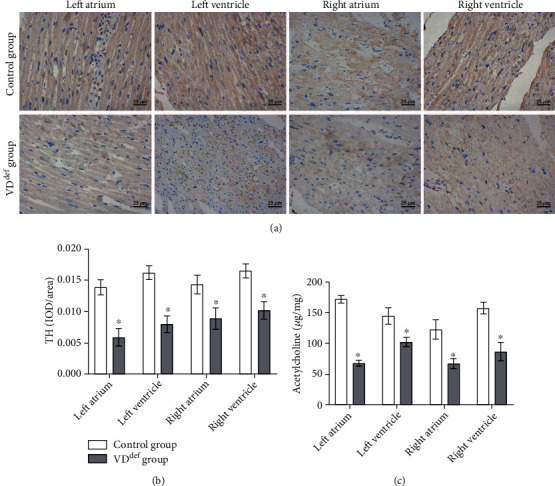
VD deficiency changes the expression of neurotransmitter in the autonomic nervous system of rats. (a) IHC was used to detect the expression of TH in rat cardiomyocytes (×400, scale bar = 25 *μ*m). (b) IOD value of TH. (c) ELISA was used to detect the level of Ach. ^∗^*P* < 0.05 vs. the control group.

To understand the effect of VD deficiency on neurotransmitters in the rat autonomic nervous system, we first analyzed the distribution of TH in different regions of the rat heart, and the IHC results showed that the expression of TH in the left atrium/ventricle and right atrium/ventricle of the VD deficiency group was remarkably lower than that in the control group (Figures 3(a) and 3(b)). The ELISA results revealed that the levels of Ach were considerably decreased in four different regions of the heart in the VD deficiency group compared with the control group (Figure 3(c)).

**Figure 4:**
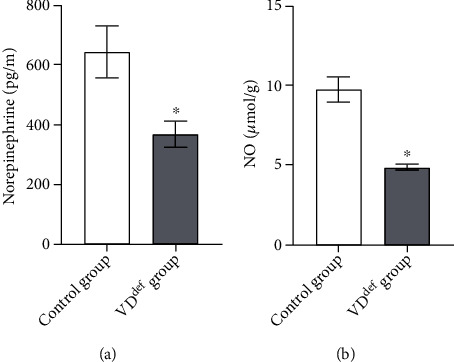
Effects of VD^def^ on local plasma hormones in the cardiac. (a) Determination of NE concentration by HPLC. (b) The concentration of NO was determined by NO kit. ^∗^*P* < 0.05 vs. the control group.

Further, we determined the levels of NE in the cardiac local plasma of rats. The HPLC results showed that the level of NE was noticeably lower in the VD deficiency group than in the control group under the motion state (Figure 4(a)). In addition, the level of NO was also reduced obviously in the VD deficiency group (Figure 4(b)).

### 3.4. Effects of VD Deficiency on Cardiac Ion Channels

**Figure 5:**
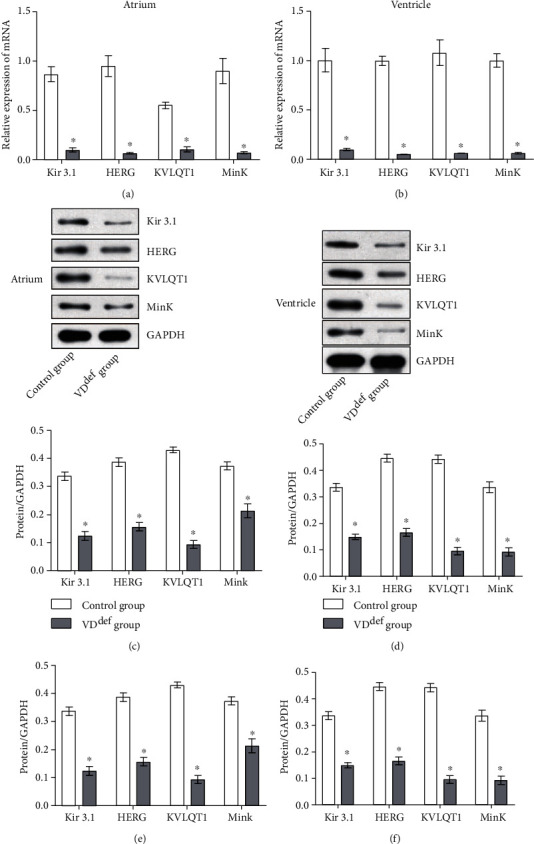
Effects of VD^def^ on ion channels in rat cardiac. (a) qRT-PCR was used to detect the expression of ion channel mRNAs (Kir3.1, HERG, KVLQT1, and Mink) in rat atria. (b) qRT-PCR was used to detect the expression of ion channel mRNA (Kir3.1, HERG, KVLQT1, Mink) in rat ventricle. (c) WB was used to detect the expression of ion channel proteins (KIR3.1, HERG, KVLQT1, and Mink) in rat atria. (d) WB was used to detect the expression of ion channel proteins (Kir3.1, HERG, KVLQT1, and Mink) in rat ventricle. ^∗^*P* < 0.05 vs. the control group.

In order to understand the effect of VD^def^ on ion channels in the cardiac of rats, qRT-PCR and WB expression analysis were performed on Kir3.1, HERG, KVLQT1, and Mink in the atrium and ventricle. Interestingly, qRT-PCR results showed that all mRNA expression levels in the ventricle and atrium of the control group were significantly higher than those in the VD^def^ group (Figure 5(a)). Similarly, WB detection showed that the expression levels of all ion channel proteins in the control group were significantly higher than those in the VD^def^ group (Figure 5(b)). Taken together, VD deficiency inhibited the normal expression of potassium ion channel proteins in rats.

## 4. Discussion

It has been proposed that the level of vitamin D is associated with cardiovascular autonomic neuropathy in patients with type I and II diabetes [20]. A study on VD levels and myocardial function revealed that a low level of VD contributes to myocardial dysfunction in adolescents [21]. However, the mechanism of influence between VD deficiency and cardiac ANS function is not clear. Therefore, the present study was devoted to investigate the influence mechanism of VD deficiency on cardiac autonomic dysfunction. In this study, we constructed a rat model of VD deficiency and measured and comparatively analyzed the physiological parameters, HRV, neurotransmitters of the ANS, cardiac local plasma hormones, and potassium channel proteins. The results showed that VD deficiency altered the physiological parameters and heart rate variability in rats and caused a decrease in the release of autonomic nervous system neurotransmitters and the secretion of cardiac local plasma hormones, and the expression of cardiac potassium channel proteins was severely impaired. However, serum creatinine, triglyceride, hemoglobin, and body weight of rats were not affected by VD deficiency.

25(OH)VD_3_ is one of the important active metabolites of VD, and its main role is to regulate the metabolism of calcium and phosphorus in the body [22]. When a vitamin D deficiency condition is present, the calcium cycle can be disrupted [23]. In this study, we found that the serum levels of calcium and phosphorus in VD-deficient rats were significantly lower than those in healthy mice. With the decrease of 25(OH)VD3, the metabolism of calcium and phosphorus in the organism was hampered. In addition, the rats fed VD-deficient diets for a longer period of time suffered from progressively slower weight gain. This suggests that VD deficiency may affect the physiological homeostasis of the organism.

Vitamin D deficiency was remarkably correlated with cardiac autonomic function as reflected by HRV. Mustafa et al. discovered that cardiac autonomic dysfunction was significantly improved in VD-deficient populations after VD supplementation [24]. Our study found that LF and HF levels were markedly lower in VD-deficient rats than in healthy rats (especially HF) in the resting state. However, the LF of VD-deficient mice was not appreciably altered in the motion state. We noticed that VLF and LF/HF were higher in VD-deficient rats in both resting and motion states. It is known that LF/HF ratio reflects the balance of ANS, where LF band represents SNS activity and HF band represents PNS activity [25]. Our results imply that VD deficiency leads to reduced parasympathetic activity and causes ANS imbalance in rats.

In the CNS region, VD modulates cardiovascular activity by coordinating other molecular mechanisms, such as neurotransmitter biosynthesis [26]. Ach is an excitatory neurotransmitter of ANS preganglionic fibers and PNS postganglionic fibers, which causes slowing of heart rate, slowing of AV node conduction, and weakening of atrial muscle contraction after acting on M-type cholinergic receptors on the cardiomyocyte membrane [27, 28]. In addition, Ach released at the endings of the vagus nerve fibers can bind to M-type cholinergic receptors of vascular smooth muscle, leading to the release of NO and vasodilation [29]. NE, known as sympathetic neurohormone, is the main transmitter of the SNS [30]. TH is a rate-limiting enzyme in the biosynthesis of NE and epinephrine [31]. VD has been proven to control the synthesis of acetylcholine and the expression of tyrosine hydroxylase [32, 33]. In our study, TH at the heart regions and Ach in the local plasma of the heart were decreased in VD-deficient rats, with a corresponding decrease in NO and NE.

Inward-rectified cardiac potassium currents include acetylcholine sensitive potassium channels (IKACH) [34]. IKACH is mainly distributed in the mammalian atrium, which affects cardiac excitability and automaticity and is involved in human heart action potential repolarization [35]. Kir3.1 is an integral part of IKACH [36]. Delayed rectifying potassium channel (*I*_k_) is the main ion stream in the diastolic automatic depolarization of pacemaker cells in the sinoatrial node, and KVLQT and HERG are its two main components [37, 38]. Mink is an important auxiliary subunit of delayed rectifying potassium channels (IKs) in cardiomyocytes and plays an important role in the normal function of IKs and is also involved in the regulation of other ion channel currents in cardiomyocytes [39]. Mink protein is an important auxiliary subunit of delayed rectifying potassium channel and plays an important role in cardiomyocyte repolarization [40–42]. By analysis at the molecular and protein levels, we found that the expression levels of potassium ion channel proteins were lower in VD-deficient rats than in normal control rats. This suggests that VD deficiency leads to impaired expression of cardiac potassium channel proteins with an impact on cardiac ANS homeostasis.

## 5. Conclusion

In conclusion, VD deficiency leads to cardiac ANS dysfunction. The imbalance in heart rate variability, impaired release and secretion of neurotransmitters and local plasma hormones in the heart, and downregulation of potassium channel protein expression caused by VD deficiency may be closely related to this dysfunction.

## Figures and Tables

**Table 1:** Primer sequence.

Gene	Sequences (5′-3′)
Mink	F GAGCCTCCTCCAAACTGGAC R TCGTCCTCACTGCTTTCCAC
HERG	F GCAGAACACCTTCCTCGACA R CATGCAGGAAATCGCAGGTG
Kir3.1	F TGAGGGACGGAAAACTCACG R GACAAGTCATCCTTTGAGCAGC
KVLQT1	F GCTGCCTTCATCTCTGCTCT R CCAGCTCCAGTGAAAAGGGA
GAPDH	F GCATCTTCTTGTGCAGTGCC R GATGGTGATGGGTTTCCCGT

**Table 2:** Comparison of physiological indexes of rats ($\bar{x}\pm \text{SD}$).

Index	Control group	VD deficiency group	*P* value
Ca (mmol/L)	3.311 ± 0.294	3.033 ± 0.268	<0.05
P (mmol/L)	15.589 ± 1.986	14.358 ± 0.862	<0.05
Creatinine (mg/dL)	23.895 ± 4.67	21.429 ± 2.417	0.0801
Triglyceride (mg/dL)	1.27 ± 0.318	1.162 ± 0.172	0.2557
Hemoglobin (g/dL)	14.494 ± 0.443	14.525 ± 0.958	0.9127
25(OH)VD_3_ (ng/mL)	4.078 ± 0.440	2.6895 ± 0.257	<0.01

## Data Availability

The data used to support the findings of this study are available from the corresponding author upon request.
